# New reactive intermediates in organic chemistry

**DOI:** 10.3762/bjoc.9.67

**Published:** 2013-03-26

**Authors:** Götz Bucher

**Affiliations:** 1 WestCHEM, School of Chemistry, University of Glasgow, Joseph-Black-Building, University Avenue, Glasgow G12 8QQ, United Kingdom

**Keywords:** reactive intermediates

Physical organic chemistry (POC) and reactive intermediate chemistry (RIC) belong to the core subjects of organic chemistry. During the 1990s and 2000s both had become increasingly less fashionable, and in many countries, the mass of active researchers working in POC and RIC was in danger of becoming subcritical. Consequently, in 2003, the Whitesides-Report on the status of chemistry in Great Britain pointed out shortcomings in POC. These shortcomings bore implications reaching beyond academia: in chemical industry, chemists with excellent skills in mechanistic organic chemistry are needed for many tasks, but an insufficient number were being educated. As a consequence of the Whitesides-Report, the Engineering and Physical Sciences Research Council of the UK (EPSRC) funded two Centres for Physical Organic Chemistry, one in Cardiff, and one in Glasgow, and launched two calls for research proposals in POC.

Research into reactive intermediates has historically been part of the work involved in product studies. In order to characterise a reactive intermediate, it had to be trapped. Thus, in order to characterise a carbene, for example, one would intercept it with an alkene and isolate the resulting cyclopropane. Using internal quenchers (molecular clocks) providing well-defined competing reactions, product studies can even yield accurate values for the kinetics of intermolecular quenching reactions of reactive intermediates. More recent techniques to characterise reactive intermediates include matrix isolation spectroscopy, where a reactive intermediate is generated in a cryogenic noble-gas matrix and hence can be studied for an extended period of time; or laser flash photolysis, where the reactive intermediate is generated by a very short pulse of laser light, and can be investigated in real time; or specialised mass-spectrometric techniques such as ion cyclotron resonance MS. Due to the exponential increase in computing power available to researchers, much of the mechanistic work nowadays is done using the tools of quantum chemistry. In particular, the various flavours of density functional theory have proven valuable in this respect, but high-level correlated methods such as CCSD(T) also have their place.

This Thematic Series of the *Beilstein Journal of Organic Chemistry* is meant to highlight recent developments in the chemistry of reactive intermediates, and to illustrate the current state of the art in a number of very varied research fields.

Götz Bucher

Glasgow, March 2013

